# Primary cytomegalovirus infection during pregnancy and congenital infection: a population-based, mother–child, prospective cohort study

**DOI:** 10.1038/s41372-021-01157-9

**Published:** 2021-07-20

**Authors:** Kyoko Shimada, Kuniaki Toriyabe, Asa Kitamura, Fumihiro Morikawa, Toshio Minematsu, Makoto Ikejiri, Shigeru Suga, Hidemi Toyoda, Keishiro Amano, Masako Kitano, Satoko Usui, Sawako Masuda, Tomoaki Ikeda

**Affiliations:** 1 Department of Obstetrics and Gynecology, Mie University Graduate School of Medicine, Tsu, Mie Japan; 2 Mie Association of Obstetricians and Gynecologists, Tsu, Mie Japan; 3 Research Center for Disease Control, Aisenkai Nichinan Hospital, Miyazaki, Japan; 4 Central Laboratory, Mie University Hospital, Tsu, Mie Japan; 5 Institute for Clinical Research, National Mie Hospital, Tsu, Mie Japan; 6 Department of Pediatrics, Mie University Graduate School of Medicine, Tsu, Mie Japan; 7 Department of Otorhinolaryngology-Head and Neck Surgery, Mie University Graduate School of Medicine, Tsu, Mie Japan; 8 Department of Otorhinolaryngology, National Mie Hospital, Tsu, Mie Japan

**Keywords:** Pathogenesis, Risk factors

## Abstract

**Objective:**

This study assessed maternal cytomegalovirus antibodies, and the occurrence of primary and congenital cytomegalovirus infections, and risk factors of congenital infection after a maternal primary infection.

**Study design:**

We included 19,435 pregnant women in Japan, who were tested for serum cytomegalovirus antibodies before 20 gestational weeks. Immunoglobulin (Ig) G avidity was evaluated in women with both IgG and IgM antibodies; tests were repeated at ≥28 gestational weeks among women without IgG and IgM antibodies.

**Result:**

Primary and congenital infections were 162 and 23 cases, respectively. The risk ratios for congenital infection were 8.18 (95% confidence interval: 2.44–27.40) in teenage versus older women, and 2.25 (95% confidence interval: 1.28–3.94) in parity ≥ 2 versus parity ≤ 1. Of 22 live birth congenital infection cases, three had abnormal neurological findings.

**Conclusion:**

We demonstrated teenage and parity ≥ 2 pregnant women as risk factors of post-primary congenital infection.

## Introduction

Cytomegalovirus (CMV) is a common pathogen that causes congenital infection, infection-related malformations, and neurological disabilities. Congenital CMV infections account for up to 10% of cases of cerebral palsy [1]. Congenital CMV is a leading cause of non-genetic sensorineural hearing loss (SNHL) at birth, accounting for 25% of all causes. Moreover, congenital CMV accounts for 25% of late-onset SNHL occurring at the age of four years [2]. Maternal CMV infections are divided into primary and non-primary infections (both occurring during pregnancy). Primary infection is the first infection a pregnant woman is exposed to. Primary infection is indicated by seroconversion or low immunoglobulin (Ig) G avidity in maternal antibody tests. Non-primary infections comprise both reinfection and reactivation of infection before pregnancy. Reinfection is caused by a CMV strain that is different from the one before pregnancy, whereas reactivation is caused by the endogen latent strain that existed before pregnancy [3].

A primary CMV infection induces a CMV-specific IgM antibody production, followed by a CMV-specific IgG antibody production. Despite the low avidity of a specific IgG antibody in the first weeks, it gradually increases after a primary CMV infection. A CMV-specific IgM antibody has a high false-positive rate, with <30% of women with positive IgM having a primary infection [4]. However, low IgG avidity is a sensitive and specific marker of primary infection [5]. Cases of IgM antibody combined with low IgG avidity are suspected of having a primary infection within the preceding 2–4 months of pregnancy [4]. The presence of the CMV IgM antibody combined with low IgG avidity is considered to have the same diagnostic value as CMV antibody seroconversion, which shows exact primary CMV infection. Lazzarotto, et al. [4] found that the incidence of fetal or newborn congenital CMV infections was very similar in both pregnant women with positive IgM antibody and low IgG avidity and those with antibody seroconversion (25.0% in women who were IgM positive with low IgG avidity and 30.3% in women with antibody seroconversion). The IgG avidity assay used in the current study appears to have a similar sensitivity for primary CMV infection as the assay used in the previous study. Ebina et al. [6] reported an 88.9% sensitivity, 96.2% specificity, 27.6% positive predictive value, and 99.8% negative predictive value, for the IgG avidity for congenital CMV infection used in the current study.

The incidence of primary CMV infection is overwhelmingly referred to in only antibody seroconversion, which occurs during pregnancy in seronegative pregnant women [7]. The incidence of primary infection during pregnancy is rarely mentioned in both sets of positive IgM and low IgG avidity and antibody seroconversion. Alternatively, for the incidence of congenital CMV infection, the incidence has been mentioned without making any distinction between the maternal primary and non-primary CMV infections.

In this population-based mother–child prospective cohort study on maternal CMV antibody screening, we demonstrated the incidence of primary and congenital CMV infection after a maternal primary infection, which occur during pregnancy. In addition, we studied the risk factors of the occurrence of congenital CMV infection after maternal primary infection.

## Subjects and methods

### Maternal CMV antibody screening program in Mie, Japan since 2013

We have been conducting maternal CMV screening programs in Mie, Japan, in the context of a population-based, observational, and prospective cohort study (UMIN000011922) since 2013. This study was conducted in accordance with the Declaration of Helsinki. We obtained ethical approval from the Clinical Research Ethics Review Committee of the Mie University Hospital (#2610) and obtained informed consent from all participants. We have previously reported the 2013–2015 maternal antibody screening program results [8]; here, we continued maternal antibody screening. Serum CMV IgG and IgM antibody tests using Seiken CMV IgG and IgM assays (Denka Seiken, Tokyo, Japan) were performed on all participants before 20 weeks of gestation. In the Seiken CMV IgG and IgM assays, the enzyme immunoassay (EIA) method was adopted. The threshold levels of both CMV IgG and IgM antibodies were determined based on the manufacturer’s protocol: CMV IgG negative, 0–1.9 EIA value; borderline, 2.0–3.9 EIA value; and positive, ≥4.0 EIA value; and CMV IgM negative, 0–0.79 index; borderline, 0.80–1.20 index; and positive, ≥1.21 index.

For the participants with IgG positive or borderline (+ or + −) and IgM positive (+) results, additional IgG avidity tests were performed, using residual serum samples from the IgG and IgM antibody tests. An Enzygnost CMV IgG assay (Siemens Healthcare Diagnostics, Tokyo, Japan) was used and the urea washing method was utilized in the Aisenkai Nichinan Hospital, Miyazaki, Japan [7]. Women with low IgG avidity results (35% or lower on the IgG avidity index) were considered as having primary infection in early pregnancy during the periconceptional period or a high risk of subsequent congenital infection; alternatively, women with high IgG avidity results (>35% of IgG avidity index) were considered as having primary infection dating >3 months pre-conception or a low risk of subsequent congenital infection. Regarding participants with IgG negative (−) and IgM negative (−) results, precautionary measures (such as avoiding close contact with saliva or urine of young children and condom usage during sexual intercourse during pregnancy) were taken to prevent primary infection. We additionally performed repeated IgG and IgM antibody tests at ≥28 weeks of gestation. Women with IgG and/or IgM seroconversion were considered as having primary infection after the first trimester of pregnancy or a high risk of subsequent congenital infection; alternatively, women with neither IgG nor IgM seroconversion had remained free from CMV infection or were seronegative with a low risk of subsequent congenital infection. For the participants with IgG (−) and IgM (+ or + −) results, we performed repeated IgG and IgM antibody tests after two or more weeks, as per the instruction manual of assays in the case of sole IgM detection. Women with IgG seroconversion were considered as having primary infection or a high risk of subsequent congenital infection; alternatively, women with no IgG seroconversion were considered as having no infection or a low risk of subsequent congenital infection. Participants with IgG (+ or + −) and IgM borderline or negative (+ − or −) results were considered as having non-primary infection or a low risk for subsequent congenital infection.

### Diagnosis of congenital CMV infection in infants whose mothers were considered as having primary infection during pregnancy

For participants considered as having primary infection during pregnancy, we collected either an amniotic fluid or a urine sample of their newborns within one week after birth. In addition, using a fresh liquid sample, we tested samples using the aforementioned real-time polymerase chain reaction (PCR) method (Mie University Hospital, Mie, Japan) [8]. Infants with CMV DNAs in the PCR method were diagnosed with congenital CMV infection. In infants with congenital infection, we performed a viral isolation method using the CMV DNAs-positive neonatal urine samples (National Mie Hospital, Mie, Japan).

Moreover, we studied the incidence (%) of congenital infection following maternal primary infection in each age group (teens, 20 s, and 30–40 s) and each parity group (para 0, para 1, and para ≥ 2). Next, we studied the risk ratio of the incidence of congenital infection.

### Neurological tests in congenitally infected infants after diagnosis of congenital CMV infection

For congenitally infected infants with abnormal findings at birth, including low birth weight, small for gestational age, microcephaly, hepatosplenomegaly, jaundice, petechia, or a “refer” result in the newborn hearing screening (NHS), we performed neurological tests, including brain magnetic resonance imaging (MRI), auditory brainstem response (ABR), and funduscopy during the neonatal period. However, infected infants who neither showed abnormal findings at birth nor a “refer” result in the NHS were neurologically tested at 18 months.

To calculate the statistical significance, we used Fisher’s exact or Chi-squared tests. A *p* < 0.05 was considered statistically significant. Analyses were performed by SAS Enterprise Guide 6.1 software (SAS Institute, Cary, NC, USA).

## Results

### Maternal CMV antibody screening

Between September 2013 and March 2017, 19,435 pregnant women participated in the maternal antibody screening program at 24 obstetric institutions in Mie, Japan, including 8469 women who were tested between September 2013 and September 2015 at 16 institutions, reported previously, and an additional 10,966 women who were tested between October 2015 and March 2017 at 24 institutions (Table 1). There were ~50 obstetrical institutions in Mie, Japan, and 49,000 deliveries during said period. We studied 40% of the women in the population as a large-scale cohort.

**Table 1:** Clinical characteristics of the participants.

	Total screened (43 months)	Sep 2013–Sep 2015 (25 months)	Oct 2015–Mar 2017(18 months)	With primary infection	With primary infection and congenital infection	With primary infection and abnormal neurological tests in congenitally-infected infants
All pregnant women	*n* = 19,435	*n* = 8469	*n* = 10,966	*n* = 162	*n* = 23	*n* = 3
Age (years)	30 (16–48)^a^	30 (16–45)^a^	30 (16–48)^a^	29 (16–41)^a^	28 (16–38)^a^	31 (30–34)^a^
teens	1.8%	1.6%	1.9%	3.7%	13.0%	0.0%
20 s	45.1%	45.2%	45.0%	46.9%	43.5%	0.0%
30 s & 40 s	53.1%	53.2%	53.1%	49.4%	43.5%	100.0%
Parity (number)	1 (0–7)^a^	1 (0–6)^a^	1 (0–7)^a^	1 (0–4)^a^	1 (0–3)^a^	1 (0–1)^a^
Para 0	47.0%	46.4%	47.4%	37.6%	43.5%	33.3%
Para 1	37.6%	38.4%	36.9%	40.8%	21.7%	66.7%
Para ≥ 2	15.5%	15.2%	15.7%	21.7%	34.8%	0.0%
GW (weeks) of IgG and IgM tests	11 (4–20)^a^	11 (4–13)^a^	11 (5–20)^a^	11 (5–17)^a^	11 (8–15)^a^	11 (11–12)^a^
≤13	87.9%	100.0%	78.5%	89.0%	91.3%	100.0%
14–17	11.2%	0.0%	19.9%	11.0%	8.7%	0.0%
18–20	0.9%	0.0%	1.5%	0.0%	0.0%	0.0%
IgG+ IgM+^b^ and low IgG avidity pregnant women	*n* = 115	*n* = 70	*n* = 45	*n* = 115	*n* = 8	*n* = 1
GW (weeks) of IgG and IgM tests	11 (5–17)^a^	11 (8–16)^a^	11 (5–17)^a^	11 (5–17)^a^	11 (5–15)^a^	11
≤13	86.2%	88.0%	82.4%	86.2%	87.5%	100.0%
14–17	13.8%	12.0%	17.6%	13.8%	12.5%	0.0%
18–20	0.0%	0.0%	0.0%	0.0%	0.0%	0.0%
IgG- IgM- pregnant women who repeated IgG and IgM tests	*n* = 4082	*n* = 1915	*n* = 2167	*n* = 47	*n* = 15	*n* = 2
GW (weeks) of repeated IgG and IgM tests	34 (28–41)^a^	34 (28–41)^a^	34 (28–41)^a^	34 (28–39)^a^	35 (28–39)^a^	36, 36
28–31	21.5%	24.4%	18.9%	23.4%	13.3%	0.0%
32–35	59.5%	54.7%	63.7%	55.3%	53.3%	0.0%
≥36	19.1%	20.9%	17.4%	21.3%	33.3%	100.0%
IgG- IgM+^b^ pregnant women who repeated IgG and IgM tests, *n* = 98	*n* = 98	*n* = 57	*n* = 41	*n* = 0	*n* = 0	*n* = 0
Interval (week) between initial and repeated IgG and IgM tests	4 (2–27)^a^	4 (2–27)^a^	4 (2–27)^a^	–	–	–
2–3	26.2%	22.8%	31.7%	–	–	–
4–5	44.0%	45.6%	41.5%	–	–	–
≥6	29.8%	31.6%	26.8%	–	–	–

*GW* gestational weeks, *Ig* immunoglobulin.

^a^Median (range).

^b^Including borderline.

A total of 19,435 pregnant women were serologically screened between September 2013 and March 2017 (8469 between September 2013 and September 2015 and 10,966 between October 2015 and March 2017). Of the 19,435 women, 162 were found with primary cytomegalovirus infection, 23 with primary infection and congenital cytomegalovirus infection, and three with primary infection and abnormal neurological tests in congenitally infected infants.

**Fig. 1:**
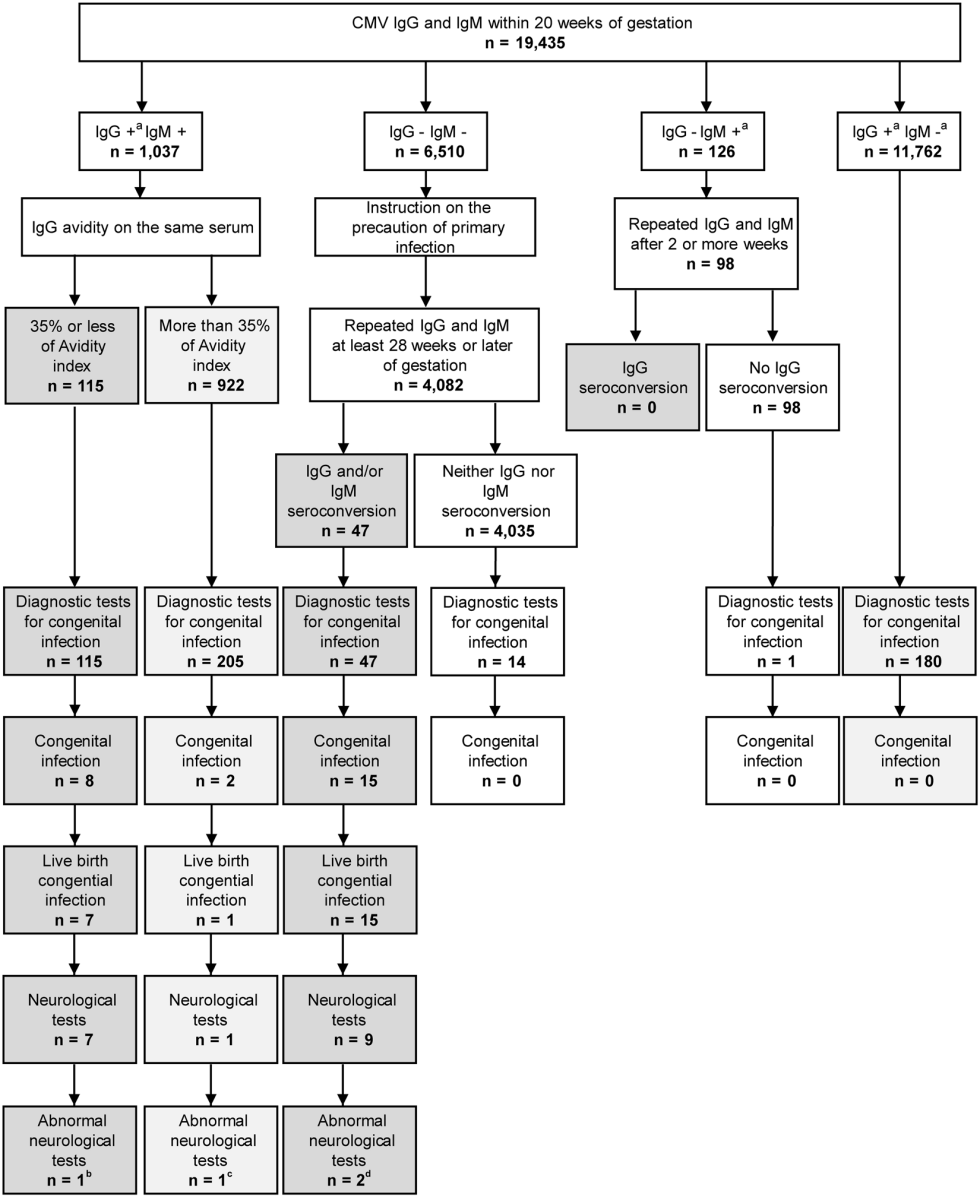
Results of maternal CMV antibody screening, diagnosis of congenital CMV infection, and infant neurological tests at approximately 18 months after birth. Primary infection was observed in 115 women with low IgG avidity and 47 with seroconversion from the initial negative IgG and negative IgM results. Congenital infection after primary infection was found in eight and 15 women with low IgG avidity and seroconversion, respectively. Live birth congenital infection after primary infection was found in seven and 15 women with low IgG avidity and seroconversion, respectively. Neurological tests were performed in seven low IgG avidity and nine seroconversion infants. Abnormal findings in neurological tests were found in one and two infants with low IgG avidity and seroconversion, respectively. ^a^Including borderline. ^b^Periventricular cysts in brain magnetic resonance imaging (MRI) and unilateral threshold elevation in auditory brainstem response (ABR). ^c^Unilateral threshold elevation in ABR. ^d^Impaired white matter intensity in brain MRI in both cases.

Out of 19,435 participants, 1037 (5.34%) had IgG (+ or + −) and IgM (+) results, of which, 115 (11.09%) showed low IgG avidity results, hence they were considered as having primary infection in early pregnancy during the periconceptional period. The other 922 women showed high IgG avidity results and were considered as having primary infection dating >3 months pre-conception. Out of 19,435 participants, 6510 (33.50%) showed IgG (−) and IgM (−) results, of which, 4082 were retested for IgG and IgM antibodies; 47 (1.15%) showed IgG and/or IgM seroconversion, being considered as having a primary infection after the first trimester of pregnancy. Out of those, 16 (0.39%) showed only IgM seroconversion while 31 (0.76%) showed IgG seroconversion; 22 showed IgG and IgM seroconversion and nine showed isolated IgG seroconversion; nevertheless, 4035 (98.85%) women showed neither IgG nor IgM seroconversion, and had remained free from CMV infection, or were seronegative. Out of the 19,435 participants, 126 (0.65%) showed IgG (−) and IgM (+ or + −) results, out of which, 98 were retested for IgG and IgM antibodies after two or more weeks. None of the 98 women showed IgG seroconversion and were considered to be without infection. Out of 19,435 participants, 11,762 (60.52%) showed IgG (+ or + −) and IgM (+ − or −) and were considered as having non-primary infections (Figs. 1,  S1).

### Congenital CMV infection in infants whose mothers were considered as having primary infection during pregnancy

We collected neonatal urine or amniotic fluid samples from 162 pregnant women considered to be primarily infected during pregnancy; 114 urine and one amniotic fluid sample from women with low IgG avidity, and 47 urine samples from women with IgG and/or IgM seroconversion from the initial IgG (−) and IgM (−) results during early pregnancy were collected. Out of the 115 low IgG avidity samples, eight (seven urine and one amniotic fluid) were positive for CMV DNAs and six (all urine) were positive for cytopathic effect in the viral isolation method. Out of 47 IgG and/or IgM seroconversion urine samples, 15 and 13 were positive for CMV DNAs and cytopathic effect, respectively, totaling 8 and 15 congenital infections in women with low IgG avidity results and in those with IgG and/or IgM seroconversion, respectively (Table 2). There was a significant difference (*p* < 0.001) in the incidence of a subsequent congenital infection between women with primary infection in early pregnancy during the periconceptional period and women with primary infection after the first trimester of pregnancy (7.0% and 31.9%, respectively). In eight pregnant women with low IgG avidity and subsequent congenital infection, the median CMV IgM titer was 7.23 index (range: 5.41–10.53 index). Keeping 100% sensitivity for the eight pregnant women, the positive predictive value for fetal congenital infection in each IgM titer level was 7.1% in the IgM titer level ≥1.21 index, 9.4% in ≥2.00 index, and 11.9% in ≥4.00 index, respectively.

**Table 2:** The number of primary, non-primary, and no infection cases with abnormal fetal echo findings, “refer” in neonatal hearing screening, and neither abnormal fetal echo findings nor “refer” in neonatal hearing screening by maternal antibody screening and congenital infection.

Maternal antibody screening and congenital infection	Abnormal fetal echo findings	“Refer” in neonatal hearing screening	Neither abnormal fetal echo findings nor “refer” in neonatal hearing screening	Total
Primary infection				
IgG+^a^ IgM+ and low IgG avidity				
With congenital infection	0	1	7	8
Without congenital infection	0	0	107	107
IgG and/or IgM seroconversion from initial IgG− IgM−				
With congenital infection	1^b^	0	14	15
Without congenital infection	0	0	32	32
Non-primary infection				
IgG+^a^ IgM+ and high IgG avidity				
With congenital infection	1^c^	0	1	2
Without congenital infection	2^b^	1	200	203
IgG+^a^ IgM−^a^				
With congenital infection	0	0	0	0
Without congenital infection	5^d^	17	158	180
No infection				
Neither IgG nor IgM seroconversion from initial IgG− IgM−				
With congenital infection	0	0	0	0
Without congenital infection	2^e^	12	0	14
No IgG seroconversion from initial IgG− IgM+^a^				
With congenital infection	0	0	0	0
Without congenital infection	0	1	0	1

*Ig* immunoglobulin.

^a^Including borderline.

^b^Fetal growth restriction.

^c^Fetal ascites.

^d^One fetal ascites and four fetal growth restriction.

^e^Fetal growth restriction and fetal ventriculomegaly.

The incidence of congenital infection following maternal primary infection was 0.86% in pregnant women in the teenage years (three out of 350), 0.11% in the 20 s (ten out of 8765), and 0.10% in the 30–40 s (ten out of 10,320), respectively. The incidence in teens was significantly higher (*p* < 0.001) and the risk ratio of the incidence of congenital infection following maternal primary infection was 8.18 (95% confidence interval: 2.44–27.40) in teens compared to the 20 s and 30–40 s age groups. Furthermore, the incidence of congenital infection following maternal primary infection was 0.11% in pregnant women of para 0 (ten out of 9115), 0.07% in para 1 (five out of 7038), and 0.27% in para ≥ 2 (eight out of 3012). The incidence in para ≥ 2 was significantly higher (*p* = 0.03), and the risk ratio of the incidence of congenital infection following maternal primary infection was 2.25 (95% confidence interval: 1.28–3.94) in para ≥ 2 compared to para 0 and para 1 (Fig. S2).

### Neurological tests in congenitally infected infants

Out of the eight congenitally infected cases in participants with low IgG avidity results, seven were live births and one was a second-trimester abortion (no abnormal fetal echo findings). All 15 congenitally infected cases in participants with seroconversion were live births. The median gestational weeks at birth of all 22 live birth cases were 38 weeks (range: 37–40 weeks) while the median birth weight was 2930 g (range: 2070–3826 g).

**Fig. 2:**
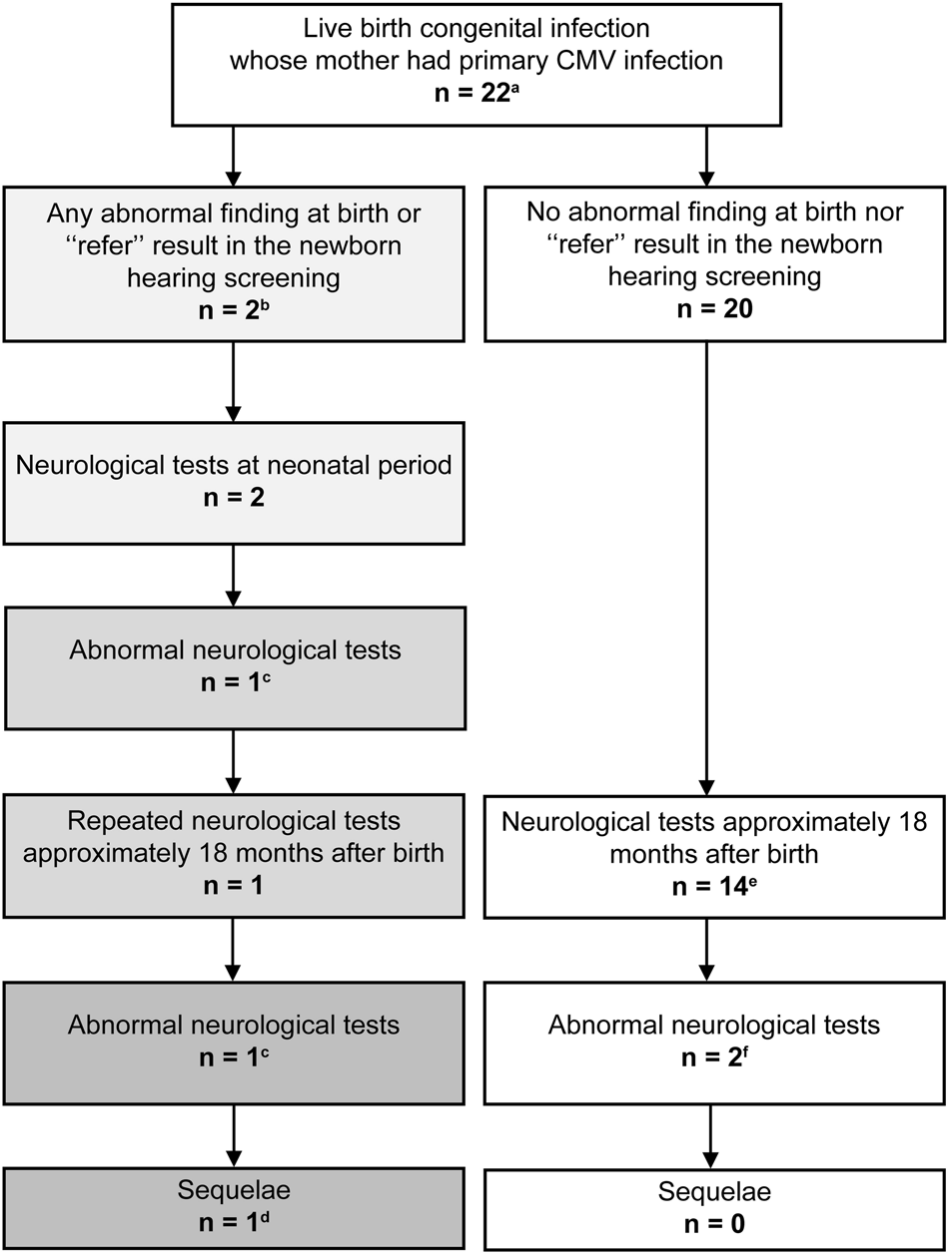
Results of infant neurological tests at approximately 18 months of age in live birth congenital CMV infection cases whose mothers had primary CMV infection (*n* = 22). ^a^Low IgG avidity (*n* = 7) and seroconversion (*n* = 15). ^b^One case from low avidity mother showed low birth weight, microcephaly, and “refer” in newborn hearing screening and the other case from seroconversion mother showed low birth weight, small for gestational age, and microcephaly. ^c^The former case showed periventricular cysts in brain magnetic resonance imaging (MRI) and unilateral threshold elevation in auditory brainstem response (ABR). ^d^Developmental delay and sensorineural hearing loss (SNHL). ^e^Six patients from low avidity mothers and eight from seroconversion mothers underwent brain MRI, ABR, and funduscopy. Another three from seroconversion mothers only underwent ABR. The remaining three did not undergo tests. ^f^Impaired white matter intensity in brain MRI in both cases.

Two out of 22 live birth cases showed abnormal findings at birth. One case from a mother with low IgG avidity (37 weeks gestation at birth, birth weight of 2244 g) showed a low birth weight, microcephaly, and a “refer” result in the NHS. The other case from a mother with seroconversion (38 weeks gestation at birth, birth weight of 2070 g) showed a low birth weight, small for gestational age, and microcephaly. They both underwent brain MRI, ABR, and funduscopy during the neonatal period. While the latter case showed normal results in all tests, the former case showed an abnormality in both brain MRI (periventricular cysts) and ABR (unilateral threshold elevation) but had normal funduscopy results. Subsequently, the former case underwent anti-viral therapy but showed developmental delay (development quotient 62) and severe unilateral SNHL. The remaining 20 out of 22 live birth cases did not show any abnormal findings at birth. Fourteen out of 20 cases without abnormal findings at birth underwent brain MRI, ABR, and funduscopy at ~18 months after birth. Two cases from mothers with seroconversion showed impaired white matter intensity in brain MRI but had normal ABR and funduscopy results (Figs. 2,  S3).

## Discussion

Serological tests diagnose maternal primary CMV infections during pregnancy, and closely examine suspicious fetal echo findings (such as hyperechogenic bowel, fetal growth restriction, or brain calcifications) and screen asymptomatic pregnant women. Recent primary infections are diagnosed by CMV IgG and IgM antibody measuring, and IgG avidity. In primary infections, a CMV IgM antibody production is induced first, followed by an IgG antibody production, which is often not detectable until at least two weeks after symptom onset [5]. The seroconversion of the CMV IgG antibody precisely means primary infection. In addition, tests regarding CMV IgG avidity are conducted to measure the IgG antibody maturity against a viral antigen to detect primary infections.

Primary infections can be confirmed by antibody seroconversion or a set of both positive IgG and IgM antibodies combined with a low IgG avidity result [9]. We studied the incidence of a set of positive IgM and low IgG avidity at early-stage pregnancy (0.59%) and seroconversion during early-to-late-stage pregnancy (0.39%) as a primary infection. A CMV IgM antibody appears and can persist for months and sometimes over a year after a primary infection. Furthermore, a CMV IgM antibody is detectable during different strain re-infection from one of the primary infections or reactivation caused by the same endogenous latent strain in the primary infection. For these reasons, the CMV IgM antibody has a high false-positive rate for primary infections, with <30% of pregnant women with positive IgM antibody having a primary infection [4]. Therefore, a diagnosis of maternal primary infection cannot be based on the production of the IgM antibody alone.

An IgG avidity test is performed to measure IgG antibody maturity against the CMV antigen. Although an IgG avidity is very low in the first weeks after primary CMV infection, it gradually increases after the primary infection. A maternal low IgG avidity result suggests a primary infection within the preceding 2–4 months [4]. Thus, a low IgG avidity result during the first trimester of pregnancy suggests a primary infection during early-stage pregnancy. Conversely, a maternal high IgG avidity result suggests a primary infection occurring more than five months earlier [9]. A high IgG avidity result during the first trimester of pregnancy suggests that primary infection occurred before conception. Moreover, a borderline IgG avidity result during the first trimester cannot exclude a primary infection either during early-stage pregnancy or the periconceptional period.

A primary CMV infection in early-stage pregnancy is usually diagnosed with a set of positive IgG and IgM antibodies and low IgG avidity results during the first trimester of pregnancy. Although an IgG avidity test is useful for diagnosing primary infections, it has limitations. Despite antibody seroconversion precisely indicating primary infection, low IgG avidity results in pregnant women do not necessarily constitute the occurrence of primary infection during pregnancy. As a diagnostic tool, a low IgG avidity result still has some pitfalls, as it may be falsely presented in past infections before conception with very low IgG antibody levels [9, 10]. Conversely, a low IgG avidity result may not be falsely presented in recent primary infections, as IgG avidity can be falsely high immediately after antibody seroconversion [11]. Therefore, exact diagnosis of maternal primary CMV infection based on the IgG avidity measurement is not perfect. In pregnant women with low IgG avidity and subsequent congenital infection, we have reported previously that the higher the CMV IgM titer, the higher the positive predictive value for congenital infection in the range of 100% sensitivity [8], which was confirmed in the current study. In pregnant women with low IgG avidity, the titer of IgM antibody was high in fetal congenital infection cases; thus, the IgM titer was considered to be useful for predicting occurrence of fetal congenital infection.

Primary infection during pregnancy is rarely mentioned in a set of positive IgM and low IgG avidity, and antibody seroconversion on a large scale; it is overwhelmingly mentioned in pregnancy antibody seroconversion in seronegative pregnant women. Hyde et al. [7] reported a 1.7% (95% confidence interval: 1.6–1.8%) incidence of antibody seroconversion during a 9-month pregnancy as in seronegative populations. In this study, we showed a 1.2% (47 IgG and/or IgM seroconversion out of 4082 seronegative pregnant women) incidence of only antibody seroconversion, mostly consistent with the reports in the literature. Kaneko et al. [12], in a Japanese cohort study, reported 0.86% of primary infection out of the total population (nine with low avidity and one with seroconversion out of 1163 pregnant women), which is similar to this study’s results, despite having a small cohort. We demonstrated that 0.98% of the maternal antibody screening cohort population is estimated to have a primary infection and the result was similar to the epidemiology in Western Europe and in the United States (~1–2% of population) (Supplementary Manuscript).

Adding to primary infection during pregnancy, the incidence of congenital infection after primary infection in the population through a large-scale maternal CMV antibody-screening cohort used in this study was 0.16% (Supplementary Manuscript, Fig. S4). We studied congenital CMV infection in pregnant women, both with a set of positive IgM and low IgG avidity at an early-stage pregnancy (0.04%) and seroconversion during early-to-late-stage pregnancy (0.12%), as primary infection during pregnancy. In the literature, the incidence of congenital CMV infection out of live births is reported to be 0.4–1.0% [13–16]; however, congenital infection has been mentioned without differentiating between maternal primary and non-primary infections during pregnancy. Recently, congenital infection has been separately reported for maternal primary and non-primary infections during pregnancy. Leruez-Ville et al. [15] reported congenital infection after maternal primary infection at 0.34% (eight out of 2378 pregnant women). Kaneko et al. [12] reported this rate at 0.26% (two out of 1163 pregnant women with IgG (+), IgM (+), and low IgG avidity and one woman with seroconversion). Tanimura et al. [17] reported a 0.14% congenital infection rate (two out of 2193 women with IgG (+), low IgG avidity, and/or IgM (+), and one woman with seroconversion). Our results were similar to the aforementioned, despite having the largest cohort, demonstrating on a large-scale cohort that 0.16% of the maternal antibody screening population is estimated to have a congenital infection after a maternal primary infection during pregnancy.

Young age and para ≥ 1 are known to be risk factors for primary CMV infection during pregnancy [18]. In this study, we studied the risk factors for congenital CMV infection after maternal primary infection and demonstrated teenage and para ≥ 2 pregnant women as risk factors of congenital infection after primary infection. Two major sources of CMV infection in pregnant women include sexual activity and direct contact with young children, with transmission occurring through direct contact with body fluids containing viable CMV. In teenage pregnant women, direct contact with semen containing CMV during sexual intercourse without condom use is thought to be a scenario of primary infection, as they rarely have children. However, in para ≥ 2 pregnant women, direct contact with urine or saliva containing CMV, of their children during pregnancy is thought to be the other scenario of primary infection.

Leruez-Ville et al. [15] reported congenital infection in pregnant women who were seronegative before pregnancy at 0.87%, eight congenital infections out of 924 women including both at early pregnancy with IgG (−) and IgM (−) results and with IgG (+), IgM (+), and low/intermediate IgG avidity results. In the current study, that occurrence was found to be lower at 0.55% (23 congenital infections out of 4197 pregnant women including 4082 with IgG (−) and IgM (−) results and 115 with IgG (+), IgM (+), and low IgG avidity results, respectively). Our education messaging provided to seronegative women to prevent primary CMV infection later in pregnancy might contribute, although we had no data relating to the number of women who acquired CMV through exposure to young children or sexual transmission in Japan. The total congenital infection in pregnant women both with primary and non-primary infection was reported to be similar between France and Japan. Total congenital infection was reported by Leruez-Ville to be 0.38% in France [15], while was reported by Koyano et al. to be 0.31% in Japan [19]. As the incidence of total congenital infection was similar between the two countries, and the incidence of congenital infection from mothers with primary infection was lower in Japan, the incidence of congenital infection from mothers with non-primary infection was thought to be higher in Japan. In fact, Tanimura et al. [17] reported that the incidence of congenital infection from mothers with non-primary infection was higher than that from those with primary infection (0.32% from non-primary and 0.14% from primary infection). Further study is needed regarding congenital infection in pregnant women with non-primary infection in Japan.

In this study, two out of nine congenital infection cases whose mothers had IgG and/or IgM seroconversion from the initial IgG (−) and IgM (−) results showed an abnormal brain MRI result. Brain lesions are thought to develop only in congenital infection cases after a maternal primary infection in the first trimester of pregnancy [18, 20–23]. Despite assumptions that pregnant women with IgG and/or IgM seroconversion from the initial IgG (−) and IgM (−) results in this study are mostly primary infections during the second or third trimester, primary infection during the first trimester may also be mixed in. The mothers of the two cases were initially IgG (−) and IgM (−) at 11 and 12 weeks of gestation, respectively; maternal primary infections might occur within the first trimester of pregnancy. If we had added antibody tests at 14 weeks of gestation, they might have shown seroconversion before late-stage pregnancy.

In conclusion, after performing a population-based, mother–child, prospective cohort study of maternal CMV antibody screening, we found primary infections during pregnancy and congenital CMV infections after maternal primary infection, and cases with abnormal results in neurological tests. In addition, we demonstrated teenage and para ≥ 2 pregnant women as risk factors of congenital infection after maternal primary infection.

## Supplementary information

Supplementary Materials
