# Supplementary material for: Primary cytomegalovirus infection during pregnancy and congenital infection: a population-based, mother–child, prospective cohort study
Source: J Perinatol. 2021 Jul 20;41(10):2474–81. doi: 10.1038/s41372-021-01157-9 (PMC8514334; doi:10.1038/s41372-021-01157-9)
Supplement: Supplementary file 1 — Supplementary Materials [file 41372_2021_1157_MOESM1_ESM.docx]

**Supplementary Materials**

**Supplementary Manuscript.** This Supplementary manuscript outlines the diagnosis and incidence of congenital infection in infants whose mothers had a non-primary infection or no infection. The results of neurological tests are also included. File format: .docx

***Estimation of the incidence of primary cytomegalovirus (CMV) infection during pregnancy and congenital infection***

We estimated the incidence of primary infection, including low IgG avidity and seroconversion, and subsequent congenital infection. For pregnant women with IgG (-) and IgM (-) results, we estimated the incidence of IgG and/or IgM seroconversion using the observed numbers, as not all women with such results were retested for these antibodies during the late pregnancy stage.

One hundred and sixty-two primary infections during pregnancy were observed, including 115 low IgG avidity and 47 seroconversion results. We also corrected for 2 428 pregnant women who were not retested for IgG and IgM antibodies during the late pregnancy stage. Subsequently, the incidence of seroconversion was estimated as 75 or 1.15% of 6 510 women with the initial IgG (-) and IgM (-) results (0.39% of all 19 435 women). Thus, the total incidence of primary infection was estimated to be 190 (0.98% of all 19 435 women, 95% confidence interval: 0.85–1.13%): 115 (0.59%) for low IgG avidity results and 75 (0.39%) for seroconversion results.

The observed incidence of congenital infection after primary infection was 23, including both eight low IgG avidity and 15 seroconversion results. We also consistently corrected for the 2 428 women who were not retested for antibodies. As a result, congenital infection after seroconversion was estimated to be 24 (0.37%) with initial IgG (-) and IgM (-) results (0.12%). Thus, the overall incidence of congenital infection after the primary infection was estimated to be 32 (0.16%, 95% confidence interval: 0.11–0.23%), with eight low IgG avidity and 24 seroconversions (Figure S4).

***Diagnosis of congenital CMV infection in some infants whose mothers were not considered as having primary infection (mothers with non-primary infection or mothers with no infection)***

For some participants who were not considered as having primary CMV infection (pregnant women with non-primary infection or those with no infection), we promptly collected urine or amniotic fluid samples in cases showing abnormal fetal echo findings (e.g., fetal ascites, growth restriction, and ventriculomegaly) or their infants showing a “refer” result in the newborn hearing screening (NHS). The antibody retest was not performed after birth using umbilical cord blood but in late pregnancy; thus, the infection during the period from antibody retest to delivery could not be identified. Therefore, a neonatal urine test was performed for abnormal fetal echo findings or "refer" result in NHS cases, even in pregnant women with no infection.

We collected both neonatal urine and amniotic fluid samples from 400 pregnant women who were considered as either having non-primary or no infection (Table 2). Six urine and two amniotic fluid samples were collected from women with non-primary infection and abnormal fetal echo findings, two urine samples were obtained from women with no infection but with abnormal fetal echo findings, 18 urine samples from women with non-primary infection and the “refer” result in the NHS, and 13 urine samples from women with no infection but with the “refer” result in the NHS. Two out of the 400 samples (one urine and one amniotic fluid sample) were positive for CMV DNAs. One urine sample tested positive for cytopathic effect in the viral isolation method.

The mothers of the two congenital infection cases had a non-primary infection. The mother of the positive case for the CMV DNAs and the cytopathic effect in the urine sample had high IgG avidity results but no abnormal fetal echo findings nor a “refer” result in the NHS [1]. The mother of the case positive for CMV DNAs in the amniotic fluid sample had high IgG avidity results and an abnormal fetal echo finding (fetal ascites) (Table 2).

***Neurological tests in congenitally infected infants after diagnosis of congenital CMV infection whose mothers were not considered as having primary infection (mothers with non-primary infection or mothers with no infection)***

One out of two congenital infection cases in participants with non-primary infection was live birth (no abnormal fetal echo findings) while the other was a second-trimester abortion (fetal ascites). The live-birth case was born at 40 weeks gestation, had a birth weight of 3 134 g and no abnormal findings at birth, and underwent brain MRI, ABR, and funduscopy 3 months after birth. This case showed abnormality only in ABR (unilateral threshold elevation) but had normal brain MRI and funduscopy results (Figure 3, Figure S3). The case also showed unilateral SNHL. We have previously reported this case as a congenital infection case whose mother had reinfection during pregnancy [1].

**References**

1. Koshizuka T, Toriyabe K, Sato Y, Ikuta K, Ikeda T, Suzutani T. Congenital cytomegalovirus infection via a re-infected mother with original antigenic sin: a case report. *Int J Infect Dis* 2018; **77**: 87–89.

**

**

**Figure S1.** Results of maternal antibody screening and diagnosis of congenital infection during the period Oct 2015-Mar 2017 (n = 10 966). File format: TIFF





**Figure S2.** Incidence (%) of congenital cytomegalovirus (CMV) infection following maternal primary CMV infection in each age group (teens, 20s, and 30–40s) and each parity group (para 0, para 1, and para ≥ 2). File format: TIFF





**Figure S3.** Results of infant neurological tests at approximately 18 months of age in live birth congenital CMV infection whose mothers had low IgG avidity (n = 7), seroconversion (n = 15), and high IgG avidity (n = 1). File format: TIFF





**Figure S4.** Flowchart of maternal antibody screening, diagnosis of congenital infection, and infant neurological tests calculated by the population number of 10 000. File format: TIFF
